# Cross-linguistic evidence for memory storage costs in filler-gap dependencies with wh-adjuncts

**DOI:** 10.3389/fpsyg.2015.01301

**Published:** 2015-09-04

**Authors:** Arthur Stepanov, Penka Stateva

**Affiliations:** Center for Cognitive Science of Language, University of Nova GoricaNova Gorica, Slovenia

**Keywords:** parsing, filler-gap dependency, thematic role, wh-adjunct, Active Filler Strategy, Slovenian

## Abstract

This study investigates processing of interrogative filler-gap dependencies in which the filler integration site or gap is not directly subcategorized by the verb. This is the case when the wh-filler is a structural adjunct such as *how* or *when* rather than subject or object. Two self-paced reading experiments in English and Slovenian provide converging cross-linguistic evidence that wh-adjuncts elicit a kind of memory storage cost similar to that previously shown in the literature for wh-arguments. Experiment 1 investigates the storage costs elicited by the adjunct *when* in Slovenian, and Experiment 2 the storage costs elicited by *how quickly* and *why* in English. The results support the class of theories of storage costs based on the metric in terms of incomplete phrase structure rules or incomplete syntactic head predictions. We also demonstrate that the endpoint of the storage cost for a wh-adjunct filler provides valuable processing evidence for its base structural position, the identification of which remains a rather murky issue in current grammatical research.

## Introduction

It has long been known that processing syntactic dependencies, in which two elements are syntactically related and linearly separated by intervening material, may be difficult for sentence comprehenders. An early study in Wanner and Maratsos (1978) showed that such difficulties arise, in particular, in processing incomplete filler-gap dependencies, in which a wh-phrase is syntactically related to the gap in the subcategorized position of the verb:

(1) Which book do you think that Colin recommended _ to the librarian?

Such long-distance dependencies are a source of syntactic complexity that the parser has to deal with over and above what is required for processing phrase structure and specific lexical items. One line of explanation for these difficulties faced by the human parser is that syntactic dependencies of this kind incur a tax on the working memory needed to temporarily store the antecedent or predictor, until a suitable element with which it can be associated is encountered in the partially processed input (Chomsky and Miller, 1963; Abney and Johnson, 1991; Gibson, 1991, 1998; Stabler, 1994; Lewis, 1996). Thus, in (1), the filler (*which* book) must somehow be temporarily stored in working memory until a suitable integration point is found. The working memory tax associated with storage cost leads to particular behavioral effects such as increased response times, or specific brain activity patterns at the neural level. Chen et al. (2005), in one of their self-paced reading experiments, manipulated the type of structure between a relative clause, where a wh-dependency is established, and a sentential complement, where it is not, as in the following examples:

**Table:** 

(2)
	a.	The announcement [that the baker from a small bakery in New York City received the award] helped the business of the owner.
	b.	The announcement [which the baker from a small bakery in New York City received ___] helped the business of the owner.

The critical region was the subject NP “the baker from a small bakery in New York City.” By hypothesis, temporarily storing the wh-filler “which” initiates an incomplete syntactic dependency and a prediction of a subcategorizing or thematic-role assigning verb to complete the dependency. Thus, assuming that both conditions otherwise involve the same amount of lexical integrations, it is predicted that the critical region in (2b) should elicit greater reading times, showing a distributed slowdown effect, as opposed to (2a) where no wh-dependency is initiated, because of the storage effect^1^. Indeed, Chen et al. (2005) observed that reading times in (2b) were greater than in (2a) (see also Gordon et al., 2002; Grodner et al., 2002 for related studies).

In the Event Related Potentials paradigm, storage costs for wh-fillers appear as a modulation of left anterior negativity (a negative voltage deflection in the frontal, often left-lateralized, regions of the scalp) spread over the region between the filler and the gap (Kluender and Kutas, 1993; King and Kutas, 1995), followed also by a P600, a positive deflection effect at the gap or pre-gap position (Kaan et al., 2000). Phillips et al. (2005) also observed that the sustained negativity persisting throughout the wh-dependency until the point of its completion is independent of the length of a filler-gap dependency, appearing both in short-distance (single clause) and long-distance (multi-clausal) wh-dependencies. The authors interpret this sustained negativity as a reflection of the cost of holding the wh-phrase in working memory. A similar pattern of Event Related Potentials was also observed in German and Japanese (Fiebach et al., 2002; Ueno and Garnsey, 2007).

In the present study, we investigate whether wh-adjuncts like *how quickly, when* and *why* elicit a similar kind of storage cost as wh-arguments do. Wh-adjuncts are notably different from wh-arguments in ways that directly affect processing. Semantically, adjuncts can never have a basic semantic type: canonically, they may function as predicates of events in the sense of event semantics (Davidson, 1980), or proposition or event modifiers in the sense of compositional semantics (Heim and Kratzer, 1998). In that capacity, wh-adjuncts are special in that their base (that is, semantically and syntactically determined) position is not predicted by subcategorization and/or thematic role assigning properties of the verb. The absence of direct association with the verb raises an a priori possibility that wh-adjuncts do not instantiate a filler-gap dependency at all: rather, they could be simply processed in their surface position. This lines up with certain grammatical theories that do not postulate syntactic displacement or dependency in the case of wh-adjuncts, as opposed to wh-arguments, and assume that wh-adjuncts are base-generated in their surface position (see, e.g., Hukari and Levine, 1995 for an overview, and the discussion below). We can then ask the following:

**Table:** 

(3)
	a.	Do wh-adjuncts instantiate filler-gap dependencies similar to wh-arguments, in the absence of thematic and/or subcategorization association with the verb?
	b.	Do all adjuncts incur similar storage costs?

As will become clear from the following discussion, we believe that the answer to (3a) is “yes,” but the answer to (3b) is most likely “no.” We do expect storage costs for (most) wh-adjuncts because their surface position must be syntactically linked to a base position linearly separated from it by intervening material. At the same time, recent advances in syntactic theory inform us that adjuncts differ with respect to their base positions. Consequently, we might expect different adjuncts to display different storage costs. The following section provides a basic overview of the major syntactic peculiarities that enter into processing considerations regarding filler-gap dependencies involving wh-adjuncts, and outlines the challenges presented by wh-adjuncts in light of the existing theories of storage costs and filler-gap dependencies in general.

### Base/integration points of wh-adjuncts

Syntactically, an adjunct is realized as a sister to an abstract syntactic node denoting the predicate that the adjunct modifies. An example of modifying an event predicate is shown in (4):

(4)
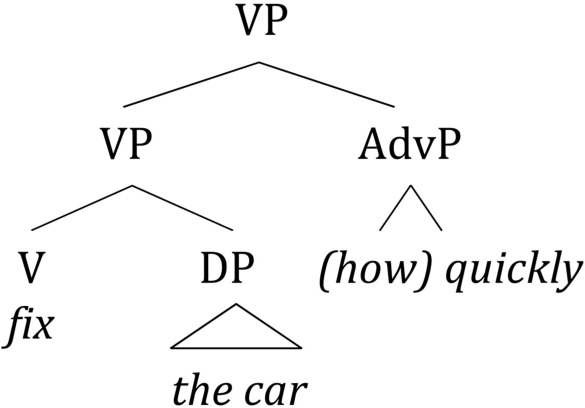

The actual attachment site of an adjunct may vary depending on the type of predicate that it modifies. Current theoretical research recognizes a multitude of positions in the syntactic structure of the sentence, where adjuncts of various semantic types may appear (e.g., Cinque, 1999). For the present purposes, we may adopt a simplified version of that typology and pinpoint at least four classes of adjuncts as in Table 1 (cf. also Ernst, 2001; Rizzi, 2001).

**Table 1:** **Grammatical typology of adjuncts and their wh-counterparts**.

**Adjunct types**	**Examples, non-wh-**	**Simplex wh-adjuncts**	**Complex wh-adjuncts**
Speaker-oriented adjuncts, or evidentials	*Clearly, obviously, fortunately, in my opinion*	–	*How obviously, how fortunately*
Reason adjuncts	*Because-*adjuncts	*Why*	*For what reason*
Subject-oriented adjuncts	*Probably*, temporal, and spatial adjuncts	*When, where*	*In which room, after which holidays*
VP-oriented adjuncts	*Quickly, often, with a hammer* etc.	*How*	*How quickly, by what means*

The order of listing the adjunct types in Table 1 roughly corresponds to their base structural position in the syntactic tree, or closeness to the root node. The attachment site of each adjunct type is determined by the corresponding phrase structure rule (e.g., S 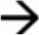 AdvP S, or VP 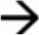 VP AdvP) and corresponds to speakers' semantic intuitions. Structurally the “highest” are speaker oriented adverbs which we will not consider in this study. The next highest position is occupied by reason adjuncts and their corresponding *wh*-counterpart *why* (see also Experiment 2 for further details), placed above the sentential S node, in the domain of COMP (or Complementizer Phrase in current syntactic terminology). This is followed by subject-oriented adjuncts that are adjoined to the S node (or Infl Phrase in many contemporary syntactic theories), not considered in this study either. Finally, VP-oriented adjuncts are adjoined to VP, thus are relatively “low” in the syntactic structure.

A major consequence of this diversity of the syntactic and semantic properties of wh-adjuncts is that, in contrast to arguments, the linear position of the adjunct in the sentence cannot be reliably predicted from the linear position of the corresponding verb and the information about the canonical word order in a language. Rather, the association of an adjunct to the verb in this case can only be *loose and indirect* [note that the lower VP constituent in (4) can syntactically be arbitrarily complex; other material can also be inserted by iterating the VP node]. In parsing, this translates into a state of affairs whereby a wh-filler cannot be reliably associated with a specific lexical stimulus, such as the verb. Rather, it may appear at an arbitrarily long distance from it^2^. With regard to VP-modifying wh-adjuncts, one can envision two possibilities as to where their integration point might lie.

The first possibility is that, despite the irrelevance of the thematic/subcategorization information, the parser follows some lexically-driven strategy to integrate the adjunct at or near the verb, similarly to wh-argument dependencies. Indeed, some grammatical models postulate a close syntactic relationship between (wh-)adjuncts and the verb outside the realm of the thematic relations. Such postulated relationship usually has a featural character: some morphosyntactic feature on the adjunct and a feature on its licensing head such as V or Infl must match or agree. In such approaches, different features may correspond to different adjuncts (Travis, 1988; Laenzlinger, 1996; Ernst, 2001). It is thus possible that the featural association of a wh-adjunct and the verb is reflected in the processing pattern, resembling or approaching the pattern of association of wh-argument fillers with corresponding verbs.

The second possibility is that the base position of the wh-adjunct (and, correspondingly, its integration point in a filler-gap dependency) requires online computation of an abstract syntactic node [cf. the lower VP in (4)] as a way of identification of the event-denoting predicate hosting the wh-adjunct, as well as some predictive information about its linear position with respect to that node. To illustrate schematically, let us assume a parsing algorithm with a storage component, using both bottom-up and top-down strategies and consulting the phrase structure module of the grammar. If such an algorithm is used to process the embedded part of a sentence like (5), the adverbial phrase *how* would be stored (possibly along with a pointer associating it with the relevant phrase structure rule; see also the discussion of the SLASH feature below) until the VP node is constructed online. Completion of the VP would trigger a subsequent application of the rule VP 
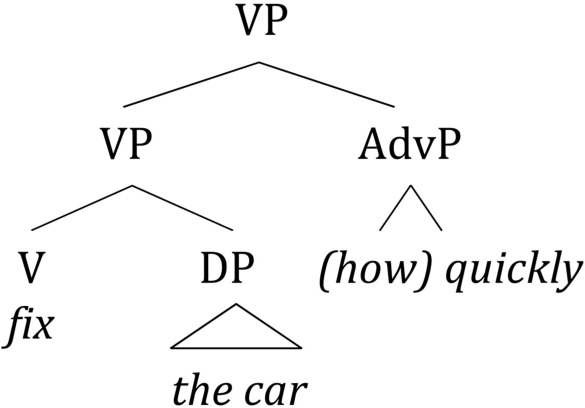
 VP AdvP, retrieving the stored phrase and integrating it at a grammatically permissible site [cf. (4)].

(5) I didn't know how John fixed the car
(6)
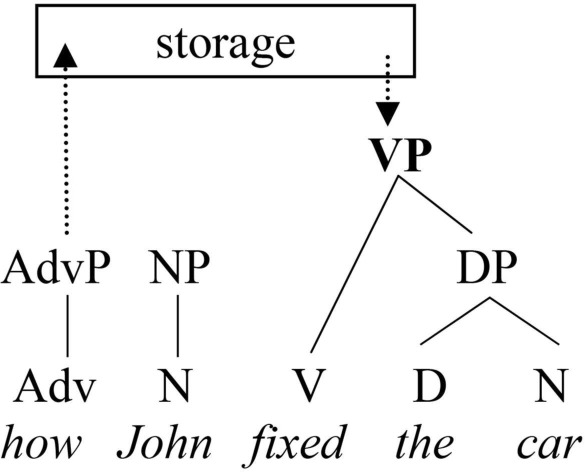

Formally, the difference between the two possibilities lies in the syntactic category of an element combining with an adjunct: a lexical head such as V^0^ vs. a phrasal category such as VP, in the correspondent grammatical rule(s) guiding integration of the adjunct during online processing. In the absence of thematic and/or subcategorization criteria for integration, syntax seems to be a major relevant cue for predicting the integration site in this case (possibly supported by other cues such as plausibility). Consequently, no lexically-based strategy would be at issue; rather, the relevant integration algorithm would have to make reference to the syntactic category information in determining the integration point (see also Gibson, 1998, 2000 concerning processing costs of structural integrations). Similar considerations apply with respect to the processing costs of temporary storage of an wh-adjunct, as discussed below.

In sum, the grammatical distribution of (wh-)adjuncts in general is much more complex than that of (wh-)arguments. Correspondingly, general processing predictions with respect to the base site of the filler-gap dependency headed by a wh-adjunct can hardly be formulated. Rather, gap predictions must be formulated item-specifically. In the absence of thematic/subcategorization information, such predictions have to take into account, at the very least, the semantic type of an adjunct, on the one hand, and the phrase structure rules generating it, on the other.

### Storing wh-adjuncts: theoretical predictions

From the storage perspective, investigating wh-adjunct dependencies is theoretically illuminating in at least two different respects. The first one concerns the role of the thematic factor in current theories of storage costs, and more generally, the role of lexically-based strategies of computation of these costs. The second regards the interaction and potential convergence of the processing and grammatical predictions concerning the endpoint of the storage costs, also in the context of the Active Filler Strategy. Below we consider these aspects in turn.

#### The lexically-based vs. syntactically-based views on storage costs

Current processing theories of incomplete filler-gap dependencies focus, explicitly or implicitly, on the issue of the temporary storage of the wh-filler, its integration into the syntactic structure of the input, or both. For instance, the memory-based accounts of filler-gap dependencies (Gibson, 1998, 2000) assume that integration and storage incur separate memory costs, and the overall processing cost of a filler gap dependency is a function of both of these measures. These accounts are based on the general idea that integrations and storage share the same pool of memory resources and that this pool of resources is limited; consequently, exceeding the set limit at some point slows down performance (Baddeley, 1990; Just and Carpenter, 1992; Lewis, 1996)^3^. From the evidence accumulating from the previous psycho- and neurolinguistic studies (see Introduction), it can be conjectured that integration costs are associated with behavioral or neurophysiological markers showing up at certain discrete points of parsing, usually at or around the predicted gap site, whereas storage costs reveal themselves as extended intervals of specific behavioral or neural response over a range of input that coincides, or is very close to, the area between the filler and the gap, in the form of a reading slowdown or increased sustained voltage deflection in the ERP signal.

The theories of storage proposed to date differ with respect to the question as to what processing units may incur a memory storage cost (see Chen et al., 2005 for review). It has been proposed that storage be measured in units such as incomplete clauses (Kimball, 1973), incomplete phrase structure rules (Yngve, 1960; Chomsky and Miller, 1963), incomplete thematic role assignments (Hakuta, 1981; Gibson, 1991), incomplete Case dependencies (Stabler, 1994), and predicted syntactic heads (Gibson, 1998, 2000). For instance, under the theory taking incomplete phrase structure rules to be relevant storage units, a center-embedded structure as in *This is the malt that the rat that the cat that the dog worried killed ate* elicits storage costs quantifiable in terms of the number of the phrase structure rules such as S 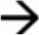 NP VP that have to be kept in memory as more embedded material is processed. Similarly, under the predicted syntactic head theory, storage is quantified in terms of the number of syntactic heads expected to complete a dependency.

We believe that investigating wh-adjunct dependencies may reliably distinguish between these theories. In particular, the theories that take storage units to be incomplete thematic role assignments (Hakuta, 1981; Gibson, 1991) predict that wh-adjuncts should not elicit storage costs, simply because there are no thematic roles associated with them that need to be stored. A similar prediction is made by the theories that take the relevant storage units to be incomplete Case dependencies (Stabler, 1994): wh-adjuncts are usually adverbials or prepositional phrases; as such, they are not subject to the Case requirement, hence no Case information needs to be stored. Thematic roles, lexical head predictions and/or Case predictions are all part of the class of theories that take lexical factors as the cornerstone of relevance when it comes to computing storage costs.

On the other hand, theories that assume that incomplete phrase structure rules are stored during processing (see above) do predict storage costs for wh-adjuncts similarly to wh-arguments. As Chen et al. (2005) point out, these theories can be adapted to handle storage costs in filler-gap wh-dependencies utilizing the analytical tools of, e.g., head-driven phrase structure grammar (Pollard and Sag, 1994) and/or generalized phrase structure grammar (Gazdar et al., 1985). In these models, the mediation between the wh-filler and the verb is achieved via the SLASH feature which may propagate across syntactic nodes or rules down to the integration site thematically associated with the verb, and thus marks the path of the wh-dependency (Pollard and Sag, 1994; Sag and Fodor, 1994). The crucial point here is that the SLASH feature is insensitive to the syntactic category of the missing constituent. Thus, all else equal, it predicts the storage costs for wh-arguments, as well as for wh-adjuncts.

Similarly, theories that compute syntactic predictions in terms of expected syntactic heads (Gibson, 1998, 2000) predict storage costs for wh-adjuncts as well as wh-arguments. In contrast to the incomplete phrase structure rule theories, the expected syntactic head model does not make direct reference to hierarchical constituent structure, but only to its lowest level of representation, the level of syntactic heads. Let us assume for the moment that the gap expectation for *how* is at the point linearly following the lower VP constituent, as in (4). For concreteness, let us also assume the algorithm for quantitative estimation of storage costs based on predicted syntactic heads, as in Gibson (2000). Consider the relevant storage costs of the sentence in (7) (concentrating on the embedded clause) that can potentially be assigned in this model, with and without the wh-adjunct, all else being equal:

**Table:** 

(7) I didn't know that/how you fixed the car yesterday							
(8)	Storage cost	Input word					
		…how	you	fixed	the	car	yesterday
		3	2	2	2	1	0
		…that	you	fixed	the	car	yesterday
		2	1	1	1	0	0

Given the nature of adjuncts as event modifiers, the storage cost at the point when *how* is processed will be 3. This reflects, in addition to the associated gap position, two more heads to describe an event (e.g., *it happened* or *John arrived*). Upon encountering the subject *you*, the storage cost value is reduced to 2, expecting a predicate and (still) a gap. At the point when *the* comes, the parser expects the noun and a gap position. Finally, at *car*, only the gap position is expected. In the non-wh version, storage costs are correspondingly reduced.

To sum up, wh-adjuncts may provide important evidence to distinguish between theories that place crucial weight on the lexical properties of fillers and those that do not. If wh-adjuncts incur storage costs, that would argue in favor of the latter type of theories. The present study seeks to provide such evidence.

#### Endpoint of the storage costs

The second interesting aspect of storage costs has to do with understanding the way storage costs for wh-adjuncts are related to the two potential grammatical possibilities for the integration site considered above. The existing theories that take storage and integration both to be active components of the working memory, largely take it for granted that the integration site marks the retrieval of the wh-filler, hence the endpoint of the storage costs. For wh-arguments, the endpoint of the storage costs at the grammatically expected point (e.g., verb for the object filler) would not be particularly surprising. For wh-adjuncts, things are not that trivial. Current grammatical theories do not always offer reliable clues as to the end-/integration point of the wh-adjunct in the syntactic structure, due to their loose and mobile syntactic character that follows from the lack of thematic and/or subcategorizational anchors. The situation gets even more complicated considering that the integration site is different for different wh-adjuncts in the same language (see footnote 2 and Experiment 2). Naturally, wh-adjunct dependencies appear somewhat more elusive for tracking with current experimental methods than their wh-argument counterparts. The endpoint of the storage costs in this situation could then provide important processing evidence for grammatical theory, to the extent that it demarcates a likely integration site.

In this respect, it is also interesting to investigate the role of the Active Filler Strategy, a parsing strategy which assigns high priority to integrating the filler at the earliest point allowed by the grammar (see Fodor, 1978; Frazier and Clifton, 1989; de Vincenzi, 1991). The Active Filler Strategy bears on the “filled gap” effect of integrating a wh-argument like subject or object with its corresponding syntactic position in the input, as in the following sentences from the self-paced reading study in Stowe (1986):

**Table:** 

(9)	a.	My brother wanted to know if Ruth will bring us home to Mom at Christmas.
	b.	My brother wanted to know [who]_i_ *G*_i_ will bring us home to Mom at Christmas.
	c.	My brother wanted to know [who]_i_ Ruth will bring ${}^{*}G_{\text{i}}$ us home to *G*_i_ at Christmas.

Longer reading times were reported at *us* in (9c) compared to (9a) and (9b). This is expected if the position of *bring* is the earliest potential position where the object wh-filler *who*, temporarily kept in the memory, can be integrated. That state of affairs causes reanalysis. In contrast, (9a) and (9b) involve no such reanalysis^4^. This and other studies investigating the Active Filler Strategy are usually based on processing verbal arguments. The interest in investigating the role of this strategy in processing wh-adjuncts consists primarily in determining (a) whether it is operative at all; and (b) if it is, what sort of evidence the parser uses in order to determine the earliest position, in the absence of thematic or lexically-oriented cues.

In the present study we report two self-paced reading experiments targeting wh-adjunct dependencies in Slovenian and English. Specifically, we focus on two examples of structurally low, VP-modifying adjuncts, as well as an example of a structurally high (reason) adjunct. Low or VP-modifying adjuncts offer a good source of evidence pertaining to research question (3a) above. Since the canonical word order in SVO languages presupposes some non-trivial distance between the occurrence of the filler and the VP in the linear representation of the interrogative sentence, a filler-gap dependency in this case can potentially be identified in parsing by a storage effect which extends across some part or all of the corresponding range in the input, much along the lines of the previous studies of storage costs incurred by wh-arguments. This is not the case with structurally high wh-adjuncts whose integration sites are likely to be close to their surface position or even identical to it (see also Section Storage Cost Predictions for *why*). Utilizing this idea, Experiment 1 aims at detecting a filler-gap dependency with the VP-modifying adjunct *kdaj* “when” in Slovenian, as well as investigating the endpoint of such dependency. Experiment 2, using English materials, addresses research question (3b) as well as (3a). It compares the storage cost patterns of the structurally low *wh-*adjunct *how quickly* and the structurally high *wh-*adjunct *why*, asking whether these processing patterns differ in a way that correlates with the syntactic and semantic properties of these two w*h*-items. This experiment also targets the endpoint of a filler-gap dependency in greater detail.

Inclusion of Slovenian in our study was justified on several grounds. Aside from the obvious benefit of expanding the empirical database of processing storage cost effects cross-linguistically, and the fact that Slovenian usually receives little attention in behavioral psycholinguistics, working with certain kinds of wh-adjunct dependencies in Slovenian turns out to be preferable as some wh-adjuncts in Slovenian are free of inherent lexical ambiguities typical of their counterparts in other languages, including English (as is the case of *when*, see below). This allows for a cleaner experimental design, avoiding potential confounds in the construction of stimuli. The present study is also the first, to our knowledge, comparing storage costs in filler-gap dependencies in two languages within the same experimental setup. Because of that, we show that storage costs elicited by wh-adjuncts are a language-independent phenomenon, a naturally expected result in the context of the general inquiry into the nature of the human parsing system.

## Experiment 1: Slovenian *kdaj* (“when”)

Experiment 1 is a self-paced reading study in which we investigate potential storage costs elicited by the structurally low, VP-modifying *wh-*adjunct *kdaj* “when” in Slovenian. If a wh-adjunct like *when* in the beginning of the sentence instantiates a filler-gap dependency as wh-arguments do, thus functioning as a filler, we may expect a storage cost effect extending across the range in the input which is commensurable with the structural distance between the filler and its corresponding gap in the VP area. The experiment tests this scenario.

In addition, Experiment 1 aims to shed light on the issue regarding the endpoint of the storage cost for *when*. As noted above, there are two main theoretical possibilities to consider with respect to this endpoint. One is that the dependency terminates at the verb, as is the case for wh-arguments. The other is that the dependency terminates at some point predicted by phrase structure rules for VP. Regarding the latter possibility, in a sentence with a transitive verb it makes sense to expect a gap at or after the relevant part of the argument structure, viz. verb plus object, is processed [cf. (4) above]. The working assumption, trivial for wh-arguments, but non-trivial for wh-adjuncts, is that the end of the storage costs (that is, a point where reading times are equalized compared to the input not involving a wh-dependency) signals the gap site. Based on the results in Chen et al. (2005) for wh-arguments, we thus expect to see a region of increased reading times to last until either the first or the second suspected gap site:

**Table:** 

(10)	a.	I didn't know when John bought (G1?) the newspaper (G2?) in the kiosk
	b.	I didn't know that John bought the newspaper in the kiosk.

If the endpoint of the storage costs is at the verb, this would support the approach to storage costs based on the featural association of the verb and the adjunct (see above). On the other hand, if the endpoint of the storage costs is at or after the direct object, this would be consistent with the phrase structural theories, as well as with the predicted syntactic head theories of storage costs.

Note that *when* in English is ambiguous. It can be used in its truly interrogative sense (cf. *Peter asked when the parcel would arrive*) or in another, related, but non-interrogative, guise (cf. *Peter left when the parcel arrived*). When used in embedded contexts, the truly interrogative version of *when* is selected by a particular class of verbs such as *ask, wonder*, or *know*. When used in its non-interrogative sense, *when* does not need to be selected at all. It is often difficult to distinguish these two usages in English and other languages which use a single lexical item for both. In Slovenian, on the other hand, the two usages of *when* are lexically disambiguated: *kdaj* is used in the respective interrogative contexts, and *ko* in non-interrogative ones. Because of that, Slovenian is an excellent choice to study the online behavior of the interrogative *when* and rule out potential confounds caused by its non-interrogative usage (which may not trigger a wh-dependency at all).

We thus concentrated on *kdaj* in Slovenian, and compared performance over the region corresponding to the argument structure (in bold) in simple embedded wh-questions such as the following (further description of the items involved is discussed in the Section Materials below):

**Table:** 

(11)	a.	Kritik je potrdil, da **je umetnik izdelal tisti** Critic is confirmed that is artist created this **koš** v svoji delavnici. basket in his workshop “The critic has confirmed that the artist created this basket in his workshop”
	b.	Kritik je potrdil, kdaj **je umetnik izdelal tisti** Critic is confirmed when is artist created this **koš** v svoji delavnici. basket ih his workshop “The critic has confirmed (the date) when the artist created this basket in his workshop”

Since in (11a) the argument structure ends at the point ***ko*š**, this is the point where we expect the storage costs to equalize with those observed at the same point in (11b). Following the critical region was either a locative PP (e.g., *v svoji delavnici* “in his workshop”) or a further optional specification of the object noun [e.g., (*koš*) *božičnih daril* “(basket) of X-mas presents”], which contained two to three words.

### Methods

#### Participants^5^

Seventy-four monolingual speakers of Slovenian from the academic communities of the University of Nova Gorica and University of Ljubljana volunteered to participate in the experiment for no material compensation. All participants were naïve to the purposes of the study.

#### Materials and methodology

Twenty-four sets of sentences, each with the two conditions described above, were carefully constructed. Since we were interested in evaluating the actual “boundaries” of the filler-gap dependencies reflected in online storage costs, the sentences in the two conditions were exactly identical except for the value of the embedded clausal head, or Complementizer: this value was either a declarative *da* “that” or interrogative *kdaj*. To control for length of a wh-dependency, all sentences were made exactly 12 words long. Each sentence began with an introductory part involving a one-word subject, a past tense auxiliary and a main verb [cf. (11)]. The main verbs were carefully chosen so that they may embed either a wh-interrogative clause, or a declarative *that-*clause. In English, typical representatives of this class of verbs are *know* and *figure out* (e.g., *I know that the guests came* vs. *I know when the guests came*; see also Experiment 2). In general, at least for *when*, the set of such ambiguously embedding verbs is much larger in Slovenian than in English, so that there was no repetition of verb between the items. The fourth word is the embedded complementizer appearing in one of the two versions outlined above. Words five through nine represent the region of interest as they minimally describe an argument structure that can be modified by *when*. The sixth word is the embedded subject, the seventh is the embedded verb and the eighth and ninth words represent the direct object, where the eighth word was always a demonstrative determiner. This was done in order to make the object structurally “heavier,” but not to the point when the complexity of its structure would potentially intervene with determination of the right boundary point. In choosing nouns used for embedded subjects and objects, as well as embedded verbs, we controlled for their plausibility and corpus frequency, for the latter using the FidaPLUS-JOS1M corpus (Erjavec et al., 2010).

The remaining three words always describe a location of the event in the form of a prepositional phrase compatible with the locative specification. The locational content of the prepositional phrase was chosen so that it would have a clear bias toward modification of the event, not of the last phrase (object). It should be also noted that Slovenian is a language where verbal clitics must always appear in the second position in the clause. The test sentences are all in past tense, whose grammatical manifestation in Slovenian requires a particular verbal clitic. That is why the second and fifth words in the test sentences are always verbal clitics, either singular or plural, depending on the grammatical number of the subject.

The target sentences were split into individualized lists balancing all factors in a Latin Square design, so that a different such list is activated for each participant. Each list was combined with 50 filler sentences of various syntactic types and of comparable length. The experimental items and fillers were thoroughly checked by a native speaker of Slovenian who is also a linguist. A complete list of target items along with their English glosses and translations is provided in Supplementary Material.

Subjects performed a self-paced reading task implemented by using the Ibex software (by Alex Drummond, http://spellout.net/ibexfarm/). We used a word-by-word centered-window presentation of stimuli. In this design, a subject initially sees two dashes in the center of the screen. By pressing the space bar, the first word in the sentence appears in place of the dashes. With each subsequent press of the space bar, the current word is replaced with the next word in the sentence, until the end of the sentence is reached. The reason we did not use the currently more popular moving window version of the self-paced reading task (see Just et al., 1982) was to rule out potential topological cues helping one to identify the left and right boundaries of a filler-gap dependency based on the positions of the relevant words or their placeholders (viz. dashes) in the linear representation of the sentence. Ruling out this possibility reinforces the scenario whereby processing filler-gap dependencies is based on the resources of working memory only, which is of primary interest from the point of view of evaluating storage costs. The order of stimulus presentation was pseudo-randomized for each participant by the experimental software and it was ensured that at least one filler intervenes between any two target items.

At the beginning of the experiment, participants were instructed to read the sentences at a natural pace and to be sure they understand what they read. To ensure that participants paid attention to the content of the reading task, half of the target items and one third of the fillers were followed by a yes-no comprehension question. Subjects were instructed to answer the question as quickly and accurately as possible. Feedback was provided when an incorrect response to a comprehension question was given, and subjects were told to take it into account as an indication to read more carefully. No feedback was given in cases of correct answer. Failure to respond within 4 s counted as an incorrect response. Before the start of the experiment, subjects read a short list of practice sentences and comprehension questions in order to familiarize themselves with the task. Each session lasted between 20 and 25 min per participant.

#### Statistical procedures

We used the same statistical procedures for all experiments in this study. To control for differences in word length across conditions as well as overall differences in participants' reading speed, a regression equation predicting reading time from word length was constructed for each participant, on the basis of all filler and experimental items (see Ferreira and Clifton, 1986). At each word position, the reading time predicted by the participant's regression equation was subtracted from the actual measured reading time to obtain a *residual reading time*. The resulting residual reading times are the dependent variable used in all analyses (Tables 2–4 also include raw reading times, to provide a more interpretable scale for the effects).

**Table 2:** **Mean (standard error) comprehension question performance in percent correct as a function of condition, by subject**.

***da***	***kdaj***
82 (1.9)	85 (1.7)

**Table 3:** **Mean residual RTs as ms/word by participants as a function of condition, for the post-COMP regions in Experiment 1, rounded to units (raw RTs in parentheses)**.

**Region**	***Da***	***Kdaj***
Cl2	−33 (421)	−18 (439)
Subj	−47 (443)	−45 (456)
V	−34 (459)	−26 (474)
Det	−30 (446)	−20 (459)
Obj	−29 (455)	−19 (473)
FU1	−9 (463)	−6 (469)
FU2	13 (502)	4 (499)
FU3	23 (520)	14 (504)

**Table 4:** **Mean residual RTs as ms/word by participants as a function of condition in Experiment 2, rounded to units (raw RTs in parentheses)**.

**Region**	***that***	***how quickly***	***why***
COMP	−29 (380)	−30 (429)	−11 (391)
Det	−32 (372)	−3 (400)	−24 (379)
N_1_	−40 (396)	−24 (412)	−43 (394)
V	−27 (412)	−15 (420)	−25 (409)
Det	3 (405)	−3 (400)	−2 (400)
N_2_	−20 (402)	−4 (410)	−15 (407)
P	−2 (394)	3 (399)	−2 (394)
N_3_	−17 (404)	−4 (417)	−8 (413)
FU1	1 (397)	10 (411)	−4 (393)
FU2	−30 (372)	−33 (370)	−33 (369)
FU3	23 (450)	14 (439)	13 (441)

For all analyses of reading time data, we used linear mixed-effects models (Baayen et al., 2008), and for question-answering data we used a logistic mixed effects model for binary data (Jaeger, 2008). The only fixed effect in our analyses was COMP(lementizer), taking values corresponding to the respective [+interrogative] or [−interrogative] complementizers, with subjects and items entered as random effects. Our constructed models utilized the maximal random effect structure with random intercepts for subjects and items and random slopes for the fixed effect term in subjects and items (Barr et al., 2013). We report *p*-values based on the likelihood-ratio test, whereby a model containing the fixed effect of interest is compared to a model that is identical in all respects except the fixed effect in question. The *p*-values are computed by treating the *t* statistic resulting from linear mixed effects analysis as approximately normally distributed (justified for datasets of our size; see Baayen et al., 2008), as also supported by visual inspection of residual plots which did not reveal any obvious deviations from homoscedasticity or normality. Analyses were performed using the lme4 package (Bates et al., 2014) in R (R Development Core Team, 2011).

#### Results

Data from five participants were omitted from all analyses because of overall poor comprehension question performance (< 67% accuracy overall). No subjects were removed on the basis of slow overall reading time (>4 standard deviations from the mean across subjects). Consequently, data from 69 subjects were used in subsequent analyses. For these subjects, reading time data from items with incorrectly answered comprehension questions were excluded from the analysis. In addition, residual reading time data points that were greater than three standard deviations from the subject mean were also excluded. This affected around 1.0% of the data overall for this experiment.

##### Comprehension questions

Overall, comprehension questions following the experimental items were answered correctly in 84% of the trials. The percentages of correct answers for each condition are presented in Table 2. A paired *t*-test revealed no significant effects [*t*_(357)_ = −0.37, *p* = 0.71]. To control for item (and subject) variability, we also fit a logistic mixed effects model and obtained similar results of COMP not being a significant predictor for the question response accuracy [$\chi_{\left(1\right)}^{2}=0.07$, *p* = 0.7935].

##### Reading times

For the primary analyses, we treated each of the 12 words within each item as its own region, according to the following schema:

(12)

**Table:** 

Kritik	je	potrdil	da/kdaj	je	umetnik	izdelal	tisti	koš	v	svoji	delavnici
critic	is	confirmed	that/when	is	artist	created	this	basket	in	his	workshop
MSubj	Cl1	MV	COMP	**Cl2**	**Subj**	**V**	**Det**	**Obj**	FU1	FU2	FU3

**Figure 1:**
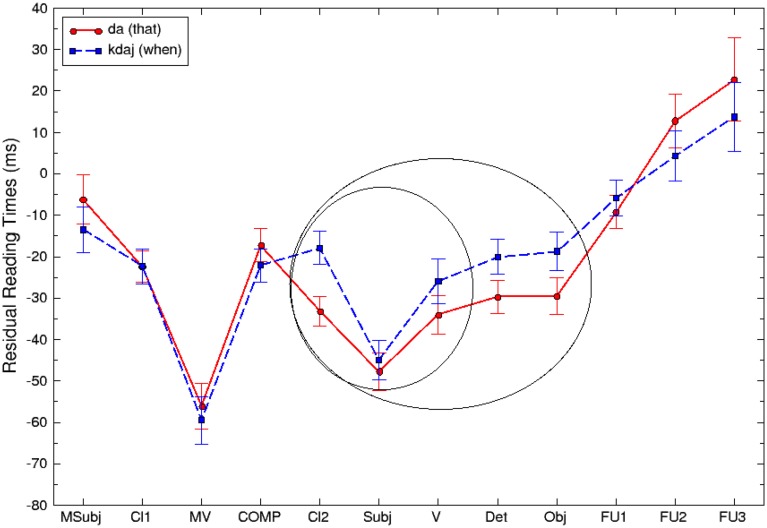
**Plot of mean (standard error) residual RTs per word by region in Experiment 1**.

Figure 1 and Table 3 show average residual reading times for each of the 12 primary regions per condition.

There was no significant effect of complementizer type in the COMP region [$\chi_{\left(1\right)}^{2}=0.71$, *p* = 0.3987]. Since COMP is selected and/or subcategorized by the matrix verb, the absence of variation suggests that the parser is equally likely to expect a [−interrogative] and [+interrogative] complementizer after the selecting verbs. This is in line with the special properties of the verbs we used in our materials: they support both types of subcategorization. This result persists across each of the four verbs used in the stimuli validating the design in terms of balancing different types of subcategorization for the same verb.

As Figure 1 and Table 3 illustrate, the interrogative *kdaj* sentences were read slower than the declarative *da* sentences in the post-COMP area until region 9 (Obj). We have defined two aggregation regions in accord with the two different kinds of theoretical predictions considered above. Recall that the first class of theories predicts that the storage costs for wh-adjuncts are distributed more or less in conformity with those for wh-arguments, that is, they are bound to the verb. Thus the first region, indicated by the smaller circle on Figure 1, spans the range of stimuli between Cl2, the first post-COMP element, and V. The second type of theory predicts that the endpoint of storage costs extends beyond the verb, namely, across the VP domain generally. Thus the second aggregation domain, indicated by the larger circle on Figure 1, includes the first and extends further to the direct object phrase, until the first follow up word FU1.

In the first aggregated region spanning the area from Cl2 until V, linear mixed models revealed a main effect of COMP, which however shows up with a marginal significance [$\chi_{\left(1\right)}^{2}=3.365$, *p* = 0.0666]. In the second, larger, aggregated region, there is a significant main effect of COMP [$\chi_{\left(1\right)}^{2}=5.3896$, *p* = 0.0203], with the relevant portions of *kdaj* clauses being read about 10 ms/word ± 3.9 ms/word (standard errors) slower than *da* clauses. We also asked whether there is a main effect of COMP specifically in the direct object area (Det + Obj) differentiating our two aggregated regions. This turned out to be the case [$\chi_{\left(1\right)}^{2}=4.427$, *p* = 0.0353], indicating that the slowdown in *kdaj* clauses persists across this particular area. Finally, regions FU1-FU3 following direct object showed no main effect of COMP, suggesting that there is no significant difference in reading times between the two conditions [$\chi_{\left(1\right)}^{2}=0.08$, *p* = 0.7795].

### Discussion

There were three main results of this experiment. The first result is that storage costs obtain for the wh-adjunct *kdaj* “when,” similarly to wh-arguments. This result holds under the assumption that the resource-consuming memory processes relevant for sentence processing involve both storage and integrations into the partially processed structure, shared in some form by most theories of storage costs up to date. Since our compared conditions only differ in the COMP value, they involve the same number of integrations. Therefore, the increased reading times in the *kdaj-*condition is likely to be attributed to a storage effect. Furthermore, the temporal span of this effect suggests that it is related specifically to processing of *kdaj* which requires additional memory resources reflected in the reading slowdown.

The second result concerns the observed time-course pattern for the storage cost effect with respect to the predictions of the two classes of theories. If the first class of theories (the filler is associated with the verb) is correct, we should expect a significant difference across the first aggregated region, but not across the second. If the second class of theories (the gap is grammatically defined as following the lowest VP constituent) is correct, then we expect the storage cost effect across the second aggregated region, including the first region as well as the area differentiating the two regions. The results indicate that the latter is the case. We have seen that the difference in reading times persists until the end of the direct object area. Under the direct (feature-) association theories predicting association of the wh-adjunct with the verb, the continuing storage effect after the verb region would remain unexplained. At the same time the observed time-course pattern of the storage cost effect is consistent with the phrase structure theories predicting a gap, or the endpoint of the storage effect, after the direct object.

An alternative interpretation of this result, suggested by a reviewer, might be that the slowdown effect over the direct object area Det+Obj is due to (spill-over) integration costs, rather than storage costs *per se*. This interpretation would then be consistent with the theories which directly associate the gap with the verb, similarly to wh-arguments, and it would confine storage costs to the pre-V region only. While this possibility cannot a priori be ruled out given the design of Experiment 1, we believe it is unlikely to be the case. Such a scenario would imply that integration of a wh-adjunct filler is just too costly: it persists through a sequence of three items (V+Det+Obj) which takes considerable time (ca. 1.5 s; see Table 3). It is true that wh-adjunct fillers are semantically more complex than wh-arguments (see Section Base/Integration Points of Wh-adjuncts). However, the alleged difficulty appears incommensurable with the relatively simple semantics of *when* as well as with the general pattern of processing filler-gap dependencies generally. In particular, no spill-over effects have been reported in the previous studies of filler-gap dependencies with wh-arguments (e.g., the ERP study of (Phillips et al., 2005) mentioned in Section Introduction observed a sustained P600 effect ending at the verb, interpreted by these authors as temporary storage cost). In addition, the fact that the post-V slowdown is restricted exactly to the entire direct object area (and not to some point before or after it) would be a suspicious coincidence under the spill-over scenario, whereas it is expected as a storage cost that conforms to the phrase structure-based theories (see above). Given these considerations, we continue to treat the direct object area as part of the relevant storage region.

The third result of Experiment 1, which stems from the second, suggests that the endpoint of the storage costs for a wh-adjunct can be a predictor of its potential gap position, or integration point. This result is important again in light of the grammatical theories that, due to excessive mobility of wh-adjuncts in the syntactic structure, often do not provide reliable diagnostics for their base position. The processing pattern is revealing in cases when the grammatical theory predicts more than one potential base position (see above), as well as in cases when it predicts a gap in a position different from the one found in a processing paradigm. An online processing study thus provides one with an efficient tool to carefully probe for the gap position in such non-trivial examples.

The results of this experiment are suggestive, but cannot be fully generalized because they are based on the processing of a single wh-item. It may be argued that the observed increased storage costs arise because of some specific lexical property of *kdaj* or, alternatively, because of some effect of interaction between this item and the syntactic structure independent of the storage cost effect. This experiment also raised an issue about the specific pattern of filler storage over the clausal subject regions. In particular, we would like to know whether the drop in the reading times is an idiosyncratic effect that occurs with specific wh-adjuncts, or representative of a more systematic pattern. Slovenian is a language whose grammar allows null/unexpressed subjects, so one could imagine a scenario where the storage cost effect expected over the subject would actually already be encoded over the preceding clitic (Cl2) region, given that this is a verbal clitic morphologically specified with the morphosemantic features of the subject (person, number, gender). Consequently, for instance, under the distance-based theory of storage costs reading the actual subject would not count toward calculating the overall storage costs for the wh-adjunct. Finally, not all wh-adjuncts are created equal. Unlike wh-arguments that are usually NPs with predicted syntactic behavior dictated by thematic considerations, wh-adjuncts may differ dramatically from each other from a syntactic point of view. There is thus an important question as to whether other wh-adjuncts elicit a similar kind of a storage cost effect, possibly correlating with their lexical, syntactic and semantic properties. Experiment 2 addresses these issues.

## Experiment 2: English *how quickly* and *why*

Experiment 1 was concerned with the wh-adjunct *when* in Slovenian, which falls under the category of VP-modifying adverbs attached relatively low in the syntactic tree (see Table 1). Experiment 2 uses English materials in a self-paced reading task and takes the investigation of storage cost effects in wh-adjunct dependencies further. The goal of this experiment was threefold. First, we wanted to replicate the Slovenian pattern of storage costs in a language in which storage costs for wh-arguments have been previously investigated in reasonable detail and at present are better understood (see Section The Lexically-based vs. Syntactically-based Views on Storage Costs), with the aim to strengthen the cross-linguistic dimension of our inquiry. English is a natural choice in this regard. Second, now that there are reasons to believe that wh-adjuncts elicit storage costs as much as wh-arguments do, the main question we ask is whether these storage costs correlate with the syntactic base position of a particular wh-adjunct, along the lines outlined in Section Endpoint of the Storage Costs. Thus in Experiment 2 we focused on the comparison between the VP modifier *how quickly* and the wh-adjunct *why*. As shown in the syntactic literature, the syntactic behavior of *why* is quite different from that of VP- modifying adjuncts, and the most robust grammatical evidence for that again comes from English (see Section Storage Cost Predictions for *why*). Since we wanted to compare the patterns of storage costs for these two modifiers, our corresponding processing predictions can therefore be better grounded in this language.

Yet another goal of Experiment 2 was to more closely investigate the integration point of the wh-adjunct in light of the relevant storage costs. Experiment 1 showed that the parser may have to wait until the direct object is parsed in order to integrate the wh-adjunct. In this respect, we were interested in the role of the Active Filler Strategy as the parser's tendency to fill the adjunct gap as soon as possible. In particular, in cases of complex direct object phrases such as *a glass of water*, does the parser wait for bottom-up evidence that the end of the direct object constituent has been reached in order to discharge the wh-adjunct, or does it do it as soon as this becomes grammatically permissible - in our example, upon encountering *a glass* (and not waiting till the end of the direct object to determine whether the phrase is complete)? This question gains particular importance in light of the proposals in the literature that derive the Active Filler Strategy from a requirement to saturate a thematic role of the wh-filler as soon as possible (Pritchett, 1992; Gibson et al., 1994; Aoshima et al., 2004). If the Active Filler Strategy is indeed a thematic-oriented strategy, it should not be relevant in the case of wh-adjunct processing. On the other hand, if the Active Filler Strategy is, in principle, independent of the thematic factor (and may or may not interact with it), then it, or some version of it, should apply in the case of wh-adjunct dependencies also, and the gap should be filled on the first grammatically permissible occasion.

### Storage cost predictions for *how quickly*

Syntactically, *how quickly* is a low, VP-modifying adjunct, and in this property it is similar to *kdaj* used in Experiment 1. Both items also have a comparable semantic status of event modifiers. Processing-wise, *how quickly* may be slightly more complex than *kdaj* because it contains an additional word^6^. For the purposes of this experiment, we will, however, treat *how quickly* as a single unit (both words were presented simultaneously to participants). Given the results of Experiment 1 using the VP adjunct *kdaj*, we expect a storage cost effect for *how quickly* across a range of input extending between the filler and the syntactically determined gap site or endpoint, which would comport with the filler's VP-modifying syntactic status. The presence of a storage effect of *how quickly* would provide further evidence strengthening the empirical validity of storage costs incurred by VP-modifying adjuncts, both on a cross-item as well as on a cross-linguistic basis.

### Storage cost predictions for *why*

Our particular interest in *why* in the present study is dictated by the growing consensus in grammatical research that the syntactic and semantic status of *why* is principally different from that of VP-modifying adjuncts like *how quickly* and *when*. Specifically, *why* has different scopal properties, different restrictions on co-occurrence with other wh-phrases, different behavior under syntactic ellipsis, sentential negation and other root phenomena (e.g., Subject-Aux inversion), compared to the other wh-adjuncts. *Why* also has semantic properties that make it different from the other *wh-*adjuncts. Whereas the latter are either event or predicate modifiers, *why* is a functor over an entire proposition (thus a question *Why did John leave the room?* has some sort of a proposition as the answer, e.g., *Because he was hurrying*, rather than a predicate modifier such as *quickly*). As an explanation for this differing behavior, it has been proposed in the syntactic literature that *why* is base-generated in its surface syntactic position in COMP at the left periphery of the sentence, or in a position very close to COMP (Bromberger, 1992; Rizzi, 2001; Stepanov and Tsai, 2008; Shlonsky and Soare, 2011). This amounts to the claim that *why* does not instantiate a filler-gap dependency in the usual sense of a long-distance dependency requiring encoding, storage and subsequent retrieval of the wh-filler. Under the standard compositional semantics approach, if *why* is a functor of propositions, *why* would then be interpreted as a sister of a syntactic node denoting a proposition, which is consistent with its base-generation at the clausal left periphery.

These syntactic and semantic accounts make a very clear prediction for a psycholinguistic study: if *why* does not initiate a filler-gap dependency, then there should be no storage effect in the case of *why*, as opposed to *how quickly*. All else equal, processing-wise, *why* is predicted to behave similarly to the complementizer *that* in a pair of sentences like (13): both expect a proposition afterwards.

**Table:** 

(13)	a.	Peter knows that John fixed the car
	b.	Peter knows why John fixed the car

We thus expect that *how quickly* and *why* will show a contrast in terms of expected storage costs. While *how quickly* should elicit storage costs similarly to Slovenian *kdaj, why* is not expected to elicit any additional storage costs compared to the *that* control. Experiment 2 tests this prediction for English.

### Methods

#### Participants

Eighty seven adult volunteers from the Glasgow community in the UK participated in this experiment voluntarily for no material compensation. All participants were recruited via email and social networking forums. All declared themselves as monolingual native speakers of English and were naïve to the purposes of the study.

#### Materials

Twenty-four sets of sentences with embedded clauses were carefully constructed^7^. Similarly to Experiment 1, the sentences in each set were exactly identical except for the value of the embedded COMP(lementizer). This time COMP takes one of the three possible values, each defining the respective condition: (1) *that*; (2) *why;* and (3) *how_quickly*. To control for the length of the wh-dependency, all sentences were made exactly 15 words long and matched by syllable structure to the best extent possible. An example is given in (14)^8^:

(14) The reporter didn't know *that/why/how_quickly* the soldier shot the panel of doctors in the hospital

Similarly to Experiment 1, each sentence begins with a four-word main clause including a two-word subject, a verbal modifier (e.g., negative *didn't* or an adverb) and a main verb in past tense. The main verbs were chosen so that they may embed either a wh-interrogative clause, or a declarative *that* clause. We chose four such ambiguously subcategorizing verbs: *know, forget, explain*, and *find_out*, which were equally represented among the set of experimental items (six instances each). The fifth word is the embedded Complementizer appearing in one of the three versions outlined above. Words six through twelve represent the main area of interest as they correspond to the verbal argument structure. The sixth and seventh words are always an embedded subject of the definite description type [the N], and the eighth is the embedded verb.

Words nine through twelve represent the direct object, which was always of the form [the N of N]. The first N is always lexically ambiguous between a standard noun and a classifier, e.g., *glass*. The second N is either a mass noun (*water*) or bare plural (*doctors*). The reason why the direct object was intentionally made structurally more complex has to do with investigating the Active Filler Strategy. If this, or similar strategy requiring gap filling as soon as possible is in place also for wh-adjuncts, we would expect the end of storage costs to occur after the first N, given a possible gap site at this location in the context of the partially processed input. Conversely, if the Active Filler strategy is not operative in the case of wh-adjuncts, then, under the assumption that phrase structure (still) guides the integration point, the gap would be expected at or after the second N. In other words, in a sentence like *I don't know how quickly John finished the drink of beer* integration of *how quickly* may occur either after *drink*, or after *beer*. The remaining three words in the sentence (words 13–15) always describe a location of the event in the form of a prepositional phrase compatible with the specification expressed by *where*.

The target sentences were split into individualized lists balancing all factors in a Latin Square design, so that a different such list is activated for each participant by the experimental software. Each such list was combined with 50 filler sentences of various syntactic types and of comparable length. The order of stimulus presentation was also pseudo-randomized separately for each participant and it was ensured that the presentation begins with a filler and that at least one filler intervenes between any two target items. A complete list of target items is provided in Supplementary Material.

The procedure was identical to the procedure in Experiment 1, except that half of the filler sentences were accompanied with a yes-no comprehension question. Computation of residual reading times and the statistical analysis procedures all followed those used in Experiment 1. In addition, Tukey's pairwise comparisons were performed on our fitted models using the *glht()* function in R's “multcomp” package (e.g., Hothorn et al., 2008).

#### Results

Ten subjects were excluded because of coding errors that led to distorting stimulus presentation in several trials. In addition, six subjects were excluded due to low comprehension question accuracy (< 67%) and/or low overall reading time (>4 standard deviations from the mean across subjects). This left the data from 71 subjects to be used in the analyses. Overall, comprehension questions were answered correctly in 88% of the trials. Residual RT data points (pooled across all regions and conditions) that were greater than three standard deviations from the mean were excluded from all analyses, affecting around 1.4% of the data overall for this experiment.

**Figure 2:**
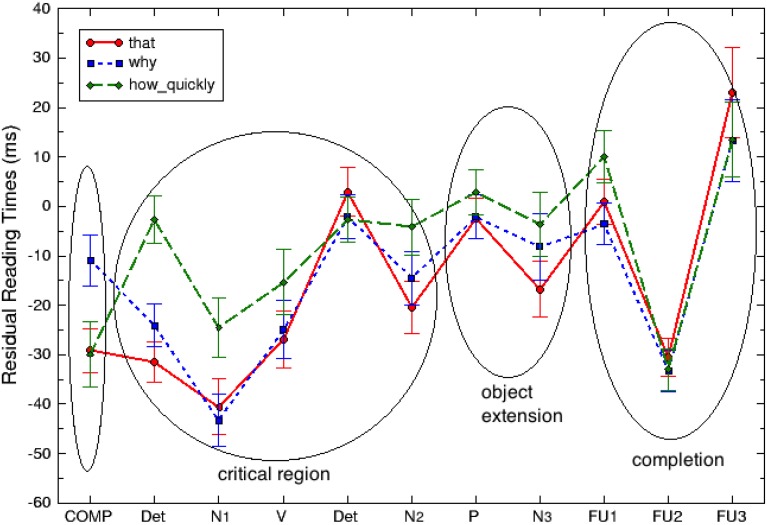
**Plot of mean (standard error) residual RTs per word by region in Experiment 2**.

For the primary analyses, we treated each of the words as its own region (omitting the main clause area). Figure 2 and Table 4 show average residual reading times for each of the 11 primary regions per condition.

We have then defined four aggregated regions of interest, as shown in (15):

(15)

**Table:** 

*that/how_quickly/why*	the	soldier	shot	the	panel	of	doctors	in	the	hospital
COMP	**Det**	**N1**	**V**	**Det**	**N2**	P	N3	FU1	FU2	FU3
	**Critical region**					Object extension		Completion		

The first region includes embedded COMP which takes one of the three condition-defining values, *that, why* or *how quickly*. Following that is the critical region which represents components of the argument structure of the embedded verb. The object extension is always an *of-*phrase (see above). The final region is a locative PP including three follow-up words. Table 5 includes estimated mean residual as well as raw reading times, illustrating a per region comparison among the three conditions.

**Table 5:** **Mean residual RTs as ms/word by participants as a function of condition, for the four aggregated regions in Experiment 2, rounded to units (raw RTs in parentheses)**.

**Aggregated region**	***that***	***how quickly***	***why***
COMP	−29 (380)	−30 (429)	−11 (397)
Critical region	−23 (397)	−9 (408)	−21 (419)
Object extension	−7 (399)	−1 (408)	−5 (403)
Completion	−2 (406)	−6 (406)	−3 (401)

At the leftmost COMP region, there is a significant variation in reading times [$\chi_{\left(2\right)}^{2}=6.2785$, *p* = 0.04331]. *Post-hoc* Tukey estimations among the pairs of conditions indicate that *why* is read slower than both *that* and *how quickly* (pair *why/that*: *z* = 2.157, *p* = 0.0783; pair *why/how_quickly*: *z* = −2.889, *p* = 0.0105; pair *that/how_quickly*: *z* = −1.146, *p* = 0.4844). The tendency to read *why* slower than *that* and *how quickly* persists across each of the four subgroups of items defined by the respective embedding verbs (*know, forget, explain, find out*) calculated separately, though does not quite reach significance within any of the subgroups (*p* > 0.05). With respect to the *why*/*that* and *how_quickly/that* pairs, this result appears to contrast with Experiment 1 where there were no notable differences in the rate of reading *da* and *kdaj*.

Moving on to the critical region, linear mixed models revealed that COMP significantly affects reading times across the range until the first N of the direct object, the first suspected integration site of the wh-adjunct dependency [$\chi_{\left(2\right)}^{2}=8.5873$, *p* = 0.01365], with the *how quickly* clauses being read about 12 ms/word ± 4.3 ms/word (standard errors) slower than the corresponding *that* clauses, and the *why* clauses virtually not affected at all with a difference of 0.3 ± 4.3 ms (standard errors) from the *that* clauses. Post-hoc pairwise Tukey comparisons confirm that the critical region of *how quickly* clauses was read significantly slower compared to *that* clauses (*z* = 2.830, *p* = 0.01284). *How quickly* clauses were also read slower than *why* clauses (*z* = 3.335, *p* = 0.00241). Finally, *why* clauses in the critical region were read with a rate similar to that of *that* clauses (*z* = −0.459, *p* = 0.89048).

In the context of estimating an endpoint of the storage cost effect and a possible impact of the Active Filler Strategy, we also asked whether the slowdown in the reading times for the *how quickly* clauses persists specifically over the part of the direct object area (Det+N2), similarly to Experiment 1. We found that, in that sub-region, *how quickly* clauses are read about 8 ms/word slower than the *that* clauses and about 5 ms/word slower than the *why* clauses. However, the effect does not reach significance [$\chi_{\left(2\right)}^{2}=2.60$, *p* = 0.27].

As Figure 2 demonstrates, *how quickly* clauses also tend to be read slower in the object extension region (the *of-*phrase), all the way up to the first follow-up word FU1. However, no main effect of COMP was estimated at the object extension region overall [$\chi_{\left(2\right)}^{2}=2.60$, *p* = 0.3491], or at each of the two word regions comprising it [region P: $\chi_{\left(2\right)}^{2}=2.6271$, *p* = 0.2687; region N_3_: $\chi_{\left(2\right)}^{2}=2.2795$, *p* = 0.3199]. Finally, at the completion region (locative PP) no significant difference in reading times across the three conditions is observed either [$\chi_{\left(2\right)}^{2}=0.6078$, *p* = 0.7379].

### Discussion

There were three main results of this experiment. The first notable result was the replication of the pattern of reading times observed in Experiment 1. In particular, *how quickly* clauses were read slower than *that* clauses in the critical region. Since, as in Experiment 1, the number of structural integrations is the same, the slowdown is likely to be due to a storage effect.

Furthermore, since both *how quickly* and Slovenian *kdaj* (“when”) share key syntactic characteristics typical for VP-modifying adjuncts, it can be concluded that such adjuncts elicit a storage effect similar to the one reported previously for wh-arguments, namely subjects and objects, and, furthermore, that this effect may be language-independent.

The second result of Experiment 2 was divergence of the patterns of the storage costs for *why* and *how quickly*. These diverging patterns would seem puzzling at face value, but they receive a natural explanation if grammatical considerations are taken into account. Since, *how quickly* needs to be kept in memory long enough to reach its integration point in the VP domain, storing it incurs a tax, much along the lines of the previous research on temporary storage of wh-arguments. At the same time, *why* does not need to be kept in memory (or it does for a very short time) because its integration site is more or less at the point where it is encountered. In this respect *why* behaves like a declarative complementizer. This result suggests that grammatical rules concerning base-generation of wh-adjuncts may serve as a reliable predictor of their storage costs, and, conversely, that observed storage cost effects provide processing evidence for the grammatical statements regarding the base position of specific (wh-)adjuncts in the syntactic structure of the sentence.

The third result, related to the second, concerns the endpoint of the storage costs for *how quickly*. Recall that inclusion of the *of-*phrase into the direct object region was motivated by our interest in the role of the Active Filler Strategy in wh-adjunct dependencies. In particular, if this or similar strategy is active, the endpoint should be observed at or around the right boundary of the critical region. If it is not active, then, under the phrase structural restrictions, we would expect the storage effect also over the object extension, the integration point then being at or right after that region.

We observed no significant difference in reading times in the direct object region, even though there is a tendency to read *how quickly* sentences slower than both *that* and *why* sentences, in that region. Thus, we did not fully replicate the result of Experiment 1 which revealed a reliable difference in the reading times between *kdaj* and *da* sentences in the direct object area. Based on the results of Experiment 1, we concluded that the parser consults the relevant phrase structural information while attempting to integrate the wh-adjunct. Given that, the reason why the English participants did not show a difference in the reading times in the direct object area following the verb could be because the grammatically permissible integration point of the adjunct *how quickly* is not the same as that for the wh-adjunct *kdaj* (“when”) namely, following the direct object. As we saw in Section Base/Integration Points of Wh-adjuncts, the grammatically licensed base position of adjuncts may be either pre-verbal or postverbal. It might be, then, that the base position of *how quickly* is actually preverbal [cf. example (ib) in fn. 2], and if so, the parser would not have to wait until the direct object in order to integrate this wh-adjunct.

The materials in Experiment 2 contained complex object noun phrases such as *the panel of doctors*. We wanted to see if the storage cost effect ends after the first, or second noun, in order to determine whether the Active Filler Strategy is operative in the case of wh-adjunct dependencies. The results of Experiment 2, namely, the absence of a reliable effect both at the first noun (in the Det+N2 region) as well as across the entire direct object area, do not permit us at this point to make a definitive conclusion in one or the other direction. Thus the possibility that the Active Filler strategy applies also in the case of wh-adjunct dependencies, cannot be ruled out.

A somewhat surprising accompanying result of Experiment 2 was a slowdown in reading the embedded *why* item itself, compared to reading times for embedded *that* and *how quickly*. The relevance of this result lies in the domain of processing subcategorized information, in particular, subcategorized complementizers. With respect to the *why/that* pair, this contrasts with Experiment 1 where there were no notable differences in the rate of reading *kdaj* vs. *da* in Slovenian. It is not clear whether verbal subcategorization for a specific question word should lead to an increased processing effort reflected in reading times, or the observed difference in reading time is simply a baseline effect. Grammatically, the verbs selected for this experiment are equally likely to select for any wh-item, or for a declarative complementizer. Previous studies on filler-gap dependencies in embedded interrogatives (notably fewer than those that investigate filler-gap dependencies in relative clauses with an invariant relativizer such as *which*) did not report any difference in reading times at the embedded COMP, e.g., between wh-arguments vs. complementizer *if* (Stowe, 1986; Lee, 2004). A number of processing factors may in principle modulate expectations for a particular subcategorization frame. For instance, studies of garden path effects suggest that verb subcategorization frequencies have an immediate effect on sentence processing (Trueswell et al., 1993; Garnsey et al., 1997; Hare et al., 2003; Snedeker and Trueswell, 2004, see also Mitchell, 1987). One may also estimate predictability of a specific verbal subcategorization by calculating its conditional probability in the context of a subcategorizer, based on corpus data (cf. Levy, 2008). Table 6 lists predictability of each of the three COMP items for each of the four embedding verbs. As Table 6 indicates, for every verb with the exception of the *explain-why* bigram the predictability drops along the continuum *that-how-why* (we take the predictability of *how* to be representative for estimating the reading time for *how_quickly*). If predictability (negatively) correlates with reading times logarithmically (Hale, 2001; Levy, 2008), then it is in principle possible that the reading times increase past some critical threshold in predictability, thus making *why* read slower. It is also possible that *why* is read slower because it is different from other wh-adjuncts, as well as from the complementizer *that*: as pointed out above, it is a functor over propositions, as opposed to VP adjuncts that are predicate modifiers, and to *that* which is just a clause-introducer. Or, again, this could be just a baseline artifact. To further clarify this issue, the follow up Experiment 3 was conducted.

**Table 6:** **Co-occurrence of the target verbs with respective COMPs, calculated as conditional probability P(COMP |VERB) = P (COMP ∩ VERB)/P(VERB), where P (COMP ∩ VERB) is a probability of the respective bigram, based on the British National Corpus (Mark Davis/Brigham Young University, http://corpus.byu.edu/bnc/)**.

**Context/COMP**	***that***	***how***	***why***
*know*	0.083	0.033	0.011
*forget*	0.097	0.017	0.002
*explain*	0.082	0.047	0.078
*find out*	0.042	0.068	0.023
MEAN	0.076	0.04125	0.0285

## Experiment 3: *why* vs. *that*

Experiment 3 had the same design as Experiment 2. This time we concentrated only on the subcategorization aspects of COMP, asking whether reading the subcategorized *why* takes additional processing effort compared to reading the embedded *that*.

### Methods

#### Participants

The procedure of subject recruitment was similar to Experiment 2. 26 English-speaking monolingual subjects volunteered to participate in this study for no material compensation.

#### Materials and procedure

Experiment 3 used a subset of the English materials used in Experiment 2. We used the same 24 target items but this time COMP only had values *that* and *why*. The rationale for choosing these items was to control for the (absence of) possible filler-gap effects at COMP, given that neither of these items instantiate a filler-gap dependency proper, as Experiment 2 has demonstrated.

Subjects saw 24 items in a pseudo-randomized order, interspersed with 52 fillers. Similarly to Experiment 2, half of the filler items were accompanied by a comprehension question.

#### Results and discussion

Overall, comprehension questions were answered correctly in 87% of the trials. No subject was excluded on the basis of comprehension accuracy or slow overall reading times (>4 standard deviations from the mean across subjects). Overall, comprehension questions were answered correctly in 87% of the trials. Residual reading time data points that were greater than three standard deviations from the mean were excluded from all analyses, affecting around 0.8% of the data for this experiment.

There was no main effect of COMP at the embedded complementizer [$\chi_{\left(1\right)}^{2}=0.768$, *p* = 0.3808]. This suggests that subcategorization does not affect the reading times of complementizers *that* and *why*. Although with only 26 participants this experiment had less statistical power than Experiment 2, this result largely corroborated that of Experiment 1. However, there is still a tendency to read *why* slower than *that*, by about 5–20 ms depending on the matrix verb [mean overall RRT (*that*) = −11 ms; mean overall RRT (*why*) = −1 ms]. Thus, if a predictability effect of the kind outlined above exists, it is very weak and requires a substantially larger statistical sample than the population size in this study to reliably reveal itself.

## General discussion

In the beginning of this article, we viewed wh-adjunct interrogatives as an important and previously under-investigated empirical ground for testing theoretical predictions pertaining to the following aspects of storage costs in filler-gap dependencies: (1) the thematic factor and the role of lexically-based strategies of computation of online storage costs; and (2) the processing and grammatical predictions concerning the endpoint of the storage costs, also in the context of the Active Filler strategy. Below we evaluate the main results of this study in light of these aspects, and point to some further issues.

### The thematic factor and the lexically-based strategies revisited

Both Experiment 1 and Experiment 2 showed a reliable storage effect related to wh-adjuncts modifying a verbal phrase (VP), that is, Slovenian *kdaj* “when” and English *how quickly*. This effect is not predicted by the class of the theories that calculate temporary storage costs in terms of the number of unassigned/incomplete thematic roles (Hakuta, 1981; Gibson, 1991), as well as in terms of the number of unassigned/incomplete Case features (Stabler, 1994). The reason is that, being non-referential syntactic entities, wh-adjuncts do not receive a thematic role from the verb, and they generally do not need Case from the verb, their Case feature being satisfied either adjunct-internally (as in the case of wh-adjunct PPs such as *on which table*), or absent at all, as in the present study. On the other hand, our results support the class of storage cost theories that do not make reference to the thematic, Case or referential status of the filler. These include theories that estimate storage costs in terms of temporarily stored incomplete phrase structure rules or their close counterpart such as the SLASH feature of HPSG (see Section The Lexically-based vs. Syntactically-based Views on Storage Costs), as well as in terms of the number of incomplete syntactic heads (Gibson, 1998, 2000). These latter theories can thus be extended to wh-argument as well as wh-adjunct dependencies.

Note that the relevant principal distinction between these two classes of theories of storage cost metric lies in the amount of theoretical weight they place on a (lexicon-oriented) internal featural specification of the filler as opposed to its (syntax-oriented) structural environment. The Case/thematic role metrics of storage costs capitalize on the thematic argument and/or the NP status of the filler. Even though theta-roles, as well as Case, have always been commonly understood as part of the syntactic computation in the grammar, it was also clear that they have a strong lexico-semantic component. In contrast, the incomplete phrase structure and incomplete syntactic head metrics of storage costs capitalize on the syntactic status of the filler, that is, its structural relation with respect to other syntactic constituents specified at the level of syntax, as in the former case, or syntactic-head driven expectations, as in the latter. Our results thus support a more syntax-oriented and less lexicon-oriented view of temporary storage costs in filler-gap dependencies^9^.

This view harmonizes with the grammatical status of (wh-)adjuncts. Since wh-adjuncts, unlike wh-arguments, are not grammatically associated with the verb directly, the integration point of a wh-adjunct in a filler-gap dependency, or its gap site, is not signaled by the relevant stimulus encountered in the input (viz. the verb). Rather, it is determined on the basis of computing an abstract syntactic node [cf. (4)] with which the wh-adjunct can be associated, in the partially processed input. It is thus reasonable to suppose that the temporarily stored information associated with the syntactically constructed host, is itself of a syntactic nature, so that this kind of computation can be performed at the same, syntactic, level. This is consistent with the modular theory of parsing (Fodor, 1978), where storing and integration can potentially be performed during the first, syntactic, pass, as well as with the interactive theories, with a qualification that no access to non-syntactic (e.g., thematic) sources of information would be needed in the case of wh-adjuncts.

### The active filler strategy revisited

The Active Filler Strategy (see Section Endpoint of the Storage Costs) was originally formulated independently from the subcategorization or theta-role assignment properties. A number of later works (e.g., Pritchett, 1992; Gibson et al., 1994; Aoshima et al., 2004) argued that the Active Filler Strategy in filler-gap dependencies reduces to the parser's need to satisfy thematic requirements of the fronted wh-phrase as soon as possible. The results of our study did not rule out the possibility that the Active Filler Strategy is operative also in wh-adjunct dependencies that are not thematically-based. If this possibility ultimately turns out to be true, that line of argument would be questioned. In this regard, we would like to briefly revisit some of the empirical evidence offered in the literature in support of recasting the Active Filler Strategy in thematic terms and consider an alternative, non-thematic interpretation of that evidence.

One empirical argument in favor of reinterpreting the Active Filler Strategy in terms of thematically-based statements comes from Aoshima et al. (2004) and is based on their experimental investigation of the Active Filler effect in Japanese, an SOV language where objects precede verbs. Aoshima et al. (2004) considered sentences with a left-scrambled wh-word that was an object of the verb in the embedded clause, as in (17) [their (7b)] which is interpreted as an embedded wh-question. Note the question word –*ka* marking the scope of that embedded question and appearing as a verbal suffix: this marker is obligatory in that context and is taken to be an interrogative complementizer:

**Table:** 

(16)	a.	Dare-ni John-wa [Mary-ga sono hon-o whom-dat John-top Mary-nom that book-acc ageta-ka] itta. gave-Q said “John said to whom Mary gave that book.”

The authors provide experimental evidence that the Japanese readers associate the scrambled wh-word with the most embedded clause of a multi-clause sentence (given the presence of the matrix subject). They argue that the wh-phrase *dare-ni* is already associated with the (bracketed) embedded clause even before the embedded verb is encountered, on the basis of a Japanese counterpart of the “filled gap” effect (Stowe, 1986, see also Section Endpoint of the Storage Costs). In particular, the readers show a surprise effect if instead of the marker–*ka* they encounter a different marker–*no* in the same context. The authors argue that if the parser's goal were simply to create a gap as soon as possible, then there would be no motivation to interpret the fronted wh-phrase inside the (most) embedded clause. Rather, the parser would posit a gap in the main clause (after the subject *John-wa*), and that gap would then be unaffected by further (embedded) structure. On the other hand, the embedded clause interpretation is expected, if the parser's objective is to satisfy thematic requirements of the verb or of the wh-phrase: the most embedded clause in an SOV language provides the first opportunity to accomplish that. In that case, the authors argue, the parser “repositions” the main clause gap as an embedded clause gap by reanalysis. On these grounds, they conclude that the Active Filler Strategy is a thematically-driven strategy (the authors also argue that the active search initiated by the parser in order to integrate the wh-phrase cannot be driven solely by the requirement to associate with the question marker; see this work for details).

The argument thus builds on the observed parser's tendency to search for the first available verb to associate with the wh-filler (see also Pritchett, 1992; Gibson et al., 1994 for similar arguments). A thematic association is indeed a natural explanation of this tendency, but, we believe, not the only one. Indeed, an association of the argument wh-filler with the verb can also be accomplished by a phrase structure rule such as V 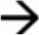 NP V, whereby the verb is a right sister of the relevant phrase. From the perspective of the parser, a lexical strategy such as “this wh-phrase must be a thematic argument of some verb, let's go and find that verb as soon as possible” is equally plausible as a syntactic strategy such as “this wh-phrase must be a structural sister of some verb, let's go and find that verb as soon as possible.” In the scenario of incremental structure building considered above, this amounts to storing the relevant phrase structure rule with an open slot (a verb in this case) in the working memory until a suitable candidate for filling in the slot is found, fully consistent with the theories of incomplete phrase structure rules. For the case of wh-arguments associated with verbs, the two strategies are virtually indistinguishable. They have the same empirical consequences, since the grammatical theory tells us that theta roles are assigned in a very local structural configuration, easily expressible with the usual machinery of phrase structure rules (e.g., Haegeman, 1994). Wh-adjuncts, however, provide a useful empirical ground for distinguishing the two strategies. The thematic/lexical strategy is not easy to restate in this case, precisely because thematic considerations are irrelevant here, whereas in the syntactic strategy, all that is needed is just to replace the relevant phrase structure rule (e.g., VP 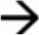 VP Adj). The syntactic strategy additionally implies that the parser is sensitive to abstract syntactic nodes as well as to lexical items, but this is a common assumption made in the parsing literature which is simply reinforced here. A different version of a syntactically-oriented strategy is de Vincenzi's (1991) re-interpretation of the Active Filler Strategy in terms of his Minimal Chain Principle: “Avoid postulating unnecessary chain members at S-structure, but do not delay required chain members” (p. 13).

### Processing evidence for the base position of Wh-adjuncts

The results from Experiment 1 and Experiment 2 are also relevant for grammatical theories regarding the base location of particular adjuncts. As mentioned in Section Base/Integration Points of Wh-adjuncts, the flexible phrase structural status of syntactic adjuncts makes it often difficult to pinpoint their base position for the purposes of explanatory syntactic analyses. This is in contrast with wh-arguments, whose base positions are usually trivially (modulo linear directionality of arguments as a parameter distinguishing, for instance, SVO from SOV languages) deduced on the basis of the linear positions of the respective predicates. With regard to wh-adjuncts, the endpoint of storage costs may provide a valuable, though admittedly indirect, processing evidence regarding these base positions. For instance, in Experiment 1 the object is a noun phrase. The end of the storage costs appears to be marked at or around the end of that noun phrase. Thus, by adjusting for the incremental character of online sentence processing, one may make an informed guess about the narrow structural area where the gap postulated by the mental grammar must lie, for each particular wh-adjunct under consideration. At the very least, the processing pattern provides us with a reasonable idea regarding directionality of the wh-adjunct gap relative to the verb. Furthermore, the absence of a continuing storage costs pattern for *why* observed in Experiment 2 is compatible with predictions of the grammatical theory regarding the non-postulation of the gap for this item. We thus have a reason to believe that the endpoint of storage costs may provide useful processing evidence for the grammatical theory of wh-adjuncts.

Overall, our results in Experiment 1 and Experiment 2 suggest that the role of the thematic factor in parsing should not be overestimated. While there is good evidence that the parser is generally sensitive to the argument structure of verbs (e.g., Rayner et al., 1983; Clifton et al., 1991; Friederici and Frisch, 2000), as far as filler-gap dependencies are concerned, the argument structure cannot be the (only) type of information that the parser makes use of during the temporary storage of the filler. Storage costs as a measure of complexity in parsing wh-adjunct dependencies suggest that phrase structure must play a role as well. In this respect, our results are consistent with the theories of integration costs employing complexity metrics of integration that do not take into account thematic information, but are based on different units of comparison, such as the number of intervening discourse referents. Our results are also consistent with the recent proposal that information from preverbal NPs may be sufficient to trigger active gap creation without having access to the verbal information including argument structure, in a kind of “hyper-active” manner (Omaki et al., 2015). In other words, the verb may not play an instrumental role in filler-gap dependencies even in the case of wh-arguments after all. In conjunction with our results on storage effects, this raises an interesting question as to whether the thematic factor can be dispensed with altogether in the processing theories of filler-gap dependencies, and replaced with the corresponding phrase structural statements. Note that in the case of wh-arguments, the thematic information is largely mirrored with the phrase structural information (this state of affairs is formalized in grammatical theory in various forms, such as “the Projection Principle,” cf. e.g., Chomsky, 1986). Thus a direct object is usually a sister of the transitive verb, and a subject is a sister of the VP. Further relevant tests for probing the role of the thematic factor independently of phrase structure may potentially include thematic and non-thematic uses of *where*, as in *where did John V the book?* in conjunction with verbs like *put* (thematic) and *see* (non-thematic), and similar constructions for other wh-adjuncts.

## Concluding remarks

The present study provided converging cross-linguistic evidence from Slovenian and English, two languages belonging to different language families, that processing filler-gap dependencies with wh-adjuncts as fillers elicit storage costs across the range of the filler-gap dependency ending approximately at the points predicted by the grammatical theories for particular adjuncts. Our findings provide evidence to the class of storage cost models that are based on computation of the number of incomplete phrase structure rules or, alternatively, the number of incomplete syntactic heads. Our results underscore the non-thematic character of storage costs, and at the same time support the principle-based approach to parsing that draws on grammatical knowledge, specifically phrase structure, as a primary source of parsing decisions (Berwick and Weinberg, 1984; Pritchett, 1988, 1991, 1992; Gibson, 1991; Gibson et al., 1994; Weinberg, 1999).

### Conflict of interest statement

The authors declare that the research was conducted in the absence of any commercial or financial relationships that could be construed as a potential conflict of interest.

## Footnotes

^1^A reviewer points out that (2a) may be compatible with a filler-gap parse up to the item *the award*, which might affect comparison of storage costs between the two sentences. This potential confound is avoided in our design of Experiment 2 with the English materials (see below).

^2^Furthermore, there is an issue of *directionality*: some adjuncts may be left-adjoined or right-adjoined, following restrictions that may have syntactic, semantic or prosodic nature (for instance, prosodically heavy adjuncts tend to be right-adjoined, see Ernst, 2001). Even within the same class of VP-adjoined adjuncts, adverbs like *quickly* allow at least two grammatically acceptable positions compatible with the sisterhood to VP (with no easily discernible difference in meaning), while other manner adverbs like *poorly* do not allow such dual positioning (Bowers, 1993):

**Table:**

(i)	a.	John fixed the car quickly
	b.	John quickly fixed the car
(ii)	a.	John fixed the car poorly
	b.	^*^John poorly fixed the car

^3^Distinguishing the integration and storage costs empirically is not a trivial task. For instance, a classic explanation of the contrast in the parsing difficulty between center-embedded structures and the corresponding right-branching structures is that the former require a greater amount of storage space as opposed to the latter, and since the amount of memory resources available for sentence processing is limited (Miller, 1956), the difficulty arises at the point when the memory capacity is exceeded (Chomsky and Miller, 1963; Abney and Johnson, 1991; Lewis, 1996). But, as Gibson (1998) notes, there exists an alternative explanation that nested structures, by their nature, always require longer distance integrations between the respective syntactic heads, hence higher processing costs, than the right-branching structures. This caveat is obviously relevant to structures manifesting filler-gap dependencies as well.

^4^Note that if *who* is ambiguous between subject and object, then the Active Filler Strategy also predicts increased reading times over the subject position (*Ruth*) in cases like (9b). In her study, Stowe found no increased reading times over the subject. However, Lee (2004) argues that a filled-gap effect appears when more material is added in between the filler and the (subject) gap, and thus sufficient time is available to the parser.

^5^The experiments in this study were carried out in accordance with the Declaration of Helsinki and the existing European and international regulations concerning ethics in research. All participants gave an informed consent prior to the beginning of testing.

^6^As pointed out at the beginning of Section Experiment 1: Slovenian kdaj (“when”), using *when* in English can potentially be confounded by its lexically ambiguous status. Other simplex wh-adjuncts in English like *where* and *how* might be subject to similar concerns if used in embedded clauses, as in our study.

^7^Preparation of the English materials and subject recruitment for Experiment 2 was implemented by Calum Riach (see Riach, 2014, supervised by the first author).

^8^A reviewer raises a concern about potentially greater pragmatic oddity/implausibility of the *how quickly* sentences as compared to the *why* counterparts, which could then lead to an increase of reading times for the former in the post-verbal (direct object) region. Even though we controlled for general plausibility upon constructing the items, we conducted an additional norming-like evaluation with the aim to see if our items are biased in this direction. We asked two native speakers of English who were not involved in creating the items, to indicate, for each item, which version out of the two sounded more natural (plausible) to them, compared to the other. Both speakers preferred the *why* reading slightly more often (by 4 and 5 items out of 24, respectively) than the *how quickly* reading. We then fit mixed effects models entering preference as a fixed factor along with COMP, asking whether preference affects the reading times, in the direct object area. We found no main effect of preference (*p* = 0.2246 and *p* = 0.6839, respectively) and no interaction with the factor COMP. This suggests that the post-verbal reading times are not affected by pragmatics/plausibility, at least in obvious ways.

^9^It should be noted that in phrase structure theories such as HPSG (see Section The lexically-based vs. Syntactically-based Views on Storage Costs) the distinction between the lexical and syntactic modules is not as clear cut as in other phrase structure theories (for instance, the transformational generative grammar). But even in that framework, the SLASH feature assigned to a lexical head (e.g., verb) is basically part of the syntactic computation establishing a relation between that head and other syntactic elements in the structure, thus the relevant processing predictions for a filler-gap dependency could arguably be made, again, on a syntactic basis.
