# Supplementary material for: Cross-linguistic evidence for memory storage costs in filler-gap dependencies with wh-adjuncts
Source: Front Psychol. 2015 Sep 4;6:1301. doi: 10.3389/fpsyg.2015.01301 (PMC4559798; doi:10.3389/fpsyg.2015.01301)
Supplement: Supplementary file 1 [file DataSheet1.PDF]

APPENDIX A: TARGET SENTENCES FOR EXPERIMENT 1 (SLOVENIAN)

Legend: acc. = Accusative Case, gen= Genitive Case, fem.=Feminine gender

(Each sentence is a source of two respective conditions)

1. Janez je videl, da/kdaj je Marko očistil tisto potovalko umazanih oblek.
   Janez is seen that/when is Marko emptied this-acc. suitcase-acc used-gen. clothes-gen.
   ”Janez saw that/when Marko emptied this suitcase of used clothes”

2. Kuharju bilo jasno, da/kdaj je pomočnik zavrgel tisto skledo očiščene
   cook-dat. was clear that/when is assistant threw-out this-acc. bowl-acc. peeled-gen.
   solate.
   lettuce-gen.
   ”It was clear to the cook that/when (his) assistant threw out this bowl of peeled lettuce”

3. Detektiv je izvedel, da/kdaj so kriminalci skrili tiste vrečke čistega kokaina.
   Detective is found out that/when are criminals hid these-acc. bags-acc. pure-gen. cocaine-gen.
   ”The detective found out that/when the criminals hid these bags of pure cocaine“

4. Arheolog je razmišljal, da/kdaj so vandali uničili tiste škatle antičnih
   Archeologist is speculated that/when are vandals destroyed these-acc. boxes-acc. ancient-gen.
   umetnin.
   arts-gen.
   ”The archeologist speculated that/when the vandals destroyed these boxes of antiquities“

5. Opazovalec je opazil, da/kdaj je družina oprala tisto posodo domačih češenj.
   Observer is noticed that/when is family washed this-acc. dish-acc. domestic-gen. cherries-gen.
   ”The observer noticed that/when the family washed this dish of home-made cherries“

6. Lastnik je pojasnil, da/kdaj so gosti ukradli tisti kovček poln denarja.
   Owner is explained that/when are guests stole this-acc. suitcase-acc. full money-gen.
   ”The owner explained that/when the guests stole this suitcase full of money”

7. Posnetek je razjasnil, da/kdaj je tat ukradel tisto košaro koruznih žemelj.
   Recording is clarified that/when is thief stole this-acc. basket-acc. corn-gen. bread-rolls-gen.
   ”The recording clarified that/when the thief stole this basket of corn bread rolls“

8. Lev je zaznal, da/kdaj je levinja odkrila tisto krdelo divjih hijen.
   Lion is found out that/when is lioness spotted this-acc. pack-acc. wild-gen. hyenas-gen.
   ”The lion found out that/when the lioness spotted this pack of wild hyenas”

9. Reporter je opisal, da/kdaj je policist razgnal tisto tolpo nevarnih
   Reporter is described that/when is police-officer dispersed this-acc. crowd-acc. dangerous-gen.
   kriminalcev.
   criminals-gen.
   ”The reporter described that/when the police officer dispersed this crowd of dangerous criminals”

10. Senator je pozabil, da/kdaj je asistent pridobil tisti vzorec volilnih okrajev.
Senator is forgot that/when is assistant obtained this-acc. list-acc. electoral-gen. districts-gen.
"The senator forgot that/when the assistant obtained this list of electoral districts"

11. Priča je razkrila, da/kdaj je osumljenec razbil tisto ampulo krvnega
Witness is revealed that/when is suspect broke this-acc. ampule-acc. bloody-gen.
vzorca.
sample-gen.
"The witness revealed that/when the suspect broke this ampule of blood sample"

12. Producent ni dojel, da/kdaj je igralka izpraznila tisto omarico zlatega nakita.
Producer not realized that/when is actress emptied this-acc. box-acc. golden-gen. jewelry-gen.
"The producer did not realize that/when the actress emptied this box of gold jewelry"

13. Borut ni razumel, da/kdaj je Metka razbila tisto vazico rdečih vrtnic.
Borut not understood that/when is Metka broke this-acc. vase-acc. red-gen. roses-gen.
"Borut did not understand that/when Metka brok this vase of red roses"

14. Raziskovalci so omenili, da/kdaj so znanstveniki razvili tiste epruvete
Researchers are mentioned that/when are scientists developed these-acc. spores-acc.
hitrega virusa.
speedy-gen virus-gen.
"The researchers mentioned that/when the scientists developed these spores of a fast virus"

15. Stražarji niso dojeli, da/kdaj je zapornik uporabljal tiste konzerve sadnega
Guards not realized that/when is prisoner used these-acc. cans-acc. fruity-gen.
kompota
compote-gen.
"The guards did not realize that/when the prisoner used these cans of fruit compote"

16. Hišnik ne ve, da/kdaj je kuharica uporabila tiste pločevinke tropskega
Housekeeper not know that/when is cook-fem. used these-acc. cans-acc. tropical-gen.
sadja.
fruit-gen.
"The housekeeper does not know that/when the (female) cook used these cans of tropical fruit"

17. Raziskovalci so odločili, da/kdaj so Maji pripravljali tiste vrče
Researchers are determined that/when are Mayans prepared these-acc. pitchers-acc.
dišeče pijače.
fragrant-gen. drink-gen.
"The researchers determined that/when the Mayans prepared these pitchers of fragrant drink"

18. Naročniki so upoštevali, da/kdaj je proizvajalec pripravil tisti nahrbtnik
Subscribers are taken-into-account that/when is manufacturer offered t this-acc. backback-acc.
taborniških oblačil.
camping-gen. clothes-gen.
"The subscribers took into account that/when the manufacturer prepared offered this backback of camping clothes"

19. Ana je dognala, da/kdaj je lastnik shranil tiste buteljke vipavske
    Ana is realized that/when is owner stored these-acc. bottles-acc. Vipavian-gen.
    penine.
    sparkling-wine-gen.
    "Ana realized that/when the owner put to storage these bottles of sparkling wine from the Vipava valley"

20. Obiskovalec je pomislil, da/kdaj je cvetličarka naredila tisti venček
    Visitor is figured out that/when is florist-fem. assembled this-acc. wreath-acc.
    belega cvetja.
    white-gen. flowers-gen.
    "The visitor figured out that/when the florist assembled this wreath of white flowers"

21. Kritik je potrdil, da/kdaj je umetnik izdelal tisti koš božičnih daril.
    Critic is made-sure that/when is artist made this-acc. basket-acc. Christmas-gen. presents-gen.
    "The critic made sure that/when the artist made this basket of Christmas presents"

22. Pilot je opazil, da/kdaj je veter razkadil tisti oblaček sivega plina.
    Pilot is noticed that/when is wind dissipated this-acc. small-cloud-acc. gray-gen. smoke-gen.
    "The pilot noticed that/when the wind dissipated this cloud of gray smoke"

23. Zavarovalnica je zagotovila, da/kdaj je naročnik napolnil tiste tubice
    Insurance-company is found out that/when is customer filled these-acc. tubes-acc.
    lepljive spojine.
    sticky compound
    "The insurance company found out that/when the customer filled these tubes of sticky compound"

24. Andrej je ugotavljal, da/kdaj je kuharica odvrgla tisti lonec domače
    Andrej is realized that/when is cook-fem. threw-away this-acc. pot-acc. homemade-acc.
    jote.
    soup
    "Andrej realized that/when the (female) cook threw away this pot of homemade soup"
