# Supplementary material for: Cross-linguistic evidence for memory storage costs in filler-gap dependencies with wh-adjuncts
Source: Front Psychol. 2015 Sep 4;6:1301. doi: 10.3389/fpsyg.2015.01301 (PMC4559798; doi:10.3389/fpsyg.2015.01301)
Supplement: Supplementary file 2 [file DataSheet2.PDF]

APPENDIX B: TARGET SENTENCES FOR EXPERIMENT 2 (ENGLISH)

COMP = *that* or *how_quickly* or *why* (each sentence is a source for three respective conditions)

1. The cleaner didn't know COMP the resident identified the chest of documents in the hall.

2. The victim didn't know COMP the murderer noticed the pool of blood in the prison.

3. The researcher didn't know COMP the soldier shot the panel of doctors in the hospital.

4. The defendant didn't know COMP the scientist instructed the teams of farmers in the summer.

5. The policeman didn't know COMP the butcher recognized the tin of meat in the shop.

6. The coach didn't know COMP the pupil indicated the block of text in the article.

7. The associate found out COMP the accountant divided the pair of charges in the bill.

8. The author found out COMP the editor recorded the techniques of painting in the books.

9. The applicant found out COMP the dentist dropped the samples of acid in the laboratory.

10. The assistant found out COMP the economist encouraged the gang of investors in the scheme.

11. The clerk found out COMP the customer approved the barrel of beer in the letter.

12. The drivers found out COMP the gardener built the structures of steel in the model.

13. The inspector foolishly forgot COMP the prisoner discovered the sheet of plastic in the cell.

14. The engineer stupidly forgot COMP the surgeon created the layer of tissue in the operation.

15. The historian regrettably forgot COMP the reporter noted the classes of errors in the list.

16. The suspect never forgot COMP the teenager dropped the basket of eggs in the forest.

17. The publisher almost forgot COMP the writer represented the code of conduct in the text.

18. The employer totally forgot COMP the colleague presented the copies of contracts in the meeting.

19. The surveyor carefully explained COMP the architect designed the suite of rooms in the college.

20. The landlord promptly explained COMP the photographer removed the box of cash in the fire.

21. The boss slowly explained COMP the journalist ordered the drink of wine in the bar.

22. The critic gradually explained COMP the guests consulted the index of records in the village.

23. The butler grudgingly explained COMP the politician proposed the network of roads in the region.

24. The spectator clearly explained COMP the referee accepted the flask of tea in the break.
